# Further Introduction of DNA Methylation (DNAm) Arrays in Regular Diagnostics

**DOI:** 10.3389/fgene.2022.831452

**Published:** 2022-07-04

**Authors:** M. M. A. M. Mannens, M. P. Lombardi, M. Alders, P. Henneman, J. Bliek

**Affiliations:** Department of Human Genetics, Amsterdam Reproduction and Development Research Institute, Amsterdam UMC, University of Amsterdam, Amsterdam, Netherlands

**Keywords:** genomic imprinting, DNAm arrays, episign, epigenome, genome diagnostics

## Abstract

Methylation tests have been used for decades in regular DNA diagnostics focusing primarily on Imprinting disorders or specific loci annotated to specific disease associated gene promotors. With the introduction of DNA methylation (DNAm) arrays such as the Illumina Infinium HumanMethylation450 Beadchip array or the Illumina Infinium Methylation EPIC Beadchip array (850 k), it has become feasible to study the epigenome in a timely and cost-effective way. This has led to new insights regarding the complexity of well-studied imprinting disorders such as the Beckwith Wiedemann syndrome, but it has also led to the introduction of tests such as EpiSign, implemented as a diagnostic test in which a single array experiment can be compared to databases with known episignatures of multiple genetic disorders, especially neurodevelopmental disorders. The successful use of such DNAm tests is rapidly expanding. More and more disorders are found to be associated with discrete episignatures which enables fast and definite diagnoses, as we have shown. The first examples of environmentally induced clinical disorders characterized by discrete aberrant DNAm are discussed underlining the broad application of DNAm testing in regular diagnostics. Here we discuss exemplary findings in our laboratory covering this broad range of applications and we discuss further use of DNAm tests in the near future.

## Introduction

Due to the Human Genome project and technological improvements, thousands of Human disorders can be diagnosed or prevented with the help of genome diagnostics, improving healthcare and reducing its costs. Genome diagnostics, however, has also confronted us with the limitations of DNA sequencing and other commonly used tests. Human health and disease are not only determined by the DNA code, but also by correct regulation of gene transcription.

Epigenetics studies this regulatory machinery. Epigenetic changes involve changes in the epigenetic modifications of chromatin, e.g., methylation of DNA or modifications of histon proteins. Also, noncoding RNAs such as microRNA or long noncoding RNAs are part of this gene transcription regulatory machinery. The modifications to the chromatin are established and recognized by proteins that either establish-, erase- or read epigenetic marks or are involved in chromatin remodeling (Bjornsson, 2015). Pathogenic mutations in these genes are associated with a large spectrum of clinical conditions often associated with intellectual disability and/or impaired neurological development.

Studying the epigenome leads to more diagnoses and better understanding of the pathogenicity of sequence variants (Aref-Eshghi et al., 2020). In addition, it leads to better understanding of environmental influences such as stress/trauma, intake of toxic substrates or inadequate nutrition in multifactorial diseases. Examples are: cardiovascular disorders, fetal alcohol syndrome, psychiatric conditions, growth malformations or the development of tumors.

Technology has moved from site specific analyses of the epigenetic status of the genome in discrete locus specific disorders to genome wide analyses of complex conditions (Ensink et al., 2021). In particular the use of the DNAm arrays, combined with improved statistics and bioinformatics approaches enables us to study the epigenome in depth, in a cost-effective way. We believe this technology has a high potential in diagnostics leading to personalized approaches in diagnostics, disease prognoses and prediction of treatment outcome.

On the other hand, there are limitations to the use of mDNA arrays in diagnostics. Carriers of Fragile X alleles for instance will not be detected until the allele becomes methylated and causes the disease. The same holds for detection of mosaic cells as seen for instance in the Beckwith Wiedemann syndrome (BWS). In addition, since gene mutations may have multiple episignatures and diseases can be multigenic, the lack of an episignature does not necessarily mean that the disorder is excluded.

### DNAm Arrays and Genomic Imprinting Disorders

During gametogenesis, fertilization, and fetal development, the epigenome drastically changes from highly unmethylated to specific DNAm patterns in specialized cells (Ischida and Moore, 2013). A special form of epigenetic gene regulation is the genomic imprinting phenomenon. For a 100 or so genes, gene regulation is determined by parental origin of the gene, i.e., paternal or maternal allele expression only. Aberrant expression of imprinted genes leads to imprinting disorders (Table 1) (Mackay and Temple, 2017) often associated with aberrant growth or even tumor development as in the Beckwith Wiedemann syndrome (Table 1).

**TABLE 1:** Overview on the twelve known imprinting disorders. Adapted from Mackay DJG, Temple IK (2017).

Imprinting Disorder OMIM	Chromosome	Associated epimutation/Reference
Transient neonatal diabetes mellitus (TNDM) 601410	6q24	*PLAGL1:alt-TSS-DMR* LOM Mackay and Temple (2010)
Silver-Russell syndrome (SRS) 180860	Chr 7 Chr 11p15	upd (7)mat *H19/IGF2*:IG:DMR LOM Eggermann 2010; Wakeling 2016
Birk–Barel syndrome 612292	Chr 8q24.3	Epimutation not yet reported (mutation in imprinted gene *KCNK9*) Barel et al (2008)
Beckwith Wiedemann syndrome (BWS) 130650	Chr 11p15	*KCNQ1OT1*:TSS-DMR LOM Chouffani et al (2010)
Kagami–Ogata syndrome (KOS14) 608149	Chr 14q32	*MEG3/DLK1*:IG-DMR GOM Ogata and Kagami (2016)
Temple syndrome (TS14) 616222	Chr 14q32	*MEG3/DLK1*:IG-DMR LOM Ioannides et al 2014; kagami et al 2017
Prader–Willi syndrome (PWS)	Chr 15q11–q13	*SNURF*:TSS-DMR GOM Buiting (2010)
Angelman syndrome (AS) 105830	Chr 15q11–q13	*SNURF*:TSS-DMR LOM Buiting (2010)
Central precocious puberty 2 (CPPB2) 615356	Chr 15q11.2	Epimutation not yet reported (mutation in imprinted gene *MKRN3*) Abreu et al (2013)
Schaaf–Yang syndrome (SYS) 615547	Chr 15q11.2	Epimutation not yet reported (mutation in imprinted gene *MAGEL2*) Fountain et al (2017)
Pseudohypoparathyroidism 1B (PHP1B) 603233	Chr 20q13	*GNAS* DMRs LOM Mantovani et al 2016; Elli et al 2016
Mulchandani–Bhoj–Conlin syndrome (MBCS) 617352	Chr 20	Epimutation not yet reported (mUPD20) Mulchandani et al (2015)

* **=** EpiSign Complete—Methylation analysis—Amsterdam UMC, genome diagnostics.

Especially in BWS, an overgrowth malformation syndrome mainly characterized by gigantism, macroglossia and exomphalos, DNA diagnostics can be complex. Two imprinted loci on the short arm of chromosome 11 are involved (i.e. imprinting center 1 (IC1) and imprinting center 2 (IC2)). Aberrant methylation can be a result of a local imprinting defect or can be caused by chromosomal abnormalities like uniparental disomies (20%), deletions (1–2%), duplication (2–4%) and translocations (rare) (Brioude et al., 2018). Aberrant methylation patterns of IC1 or IC2 lead to aberrant expression of genes associated with BWS. The best practice guidelines for BWS diagnostics have been published by Brioude et al. (2018), Such aberrant methylation patterns, consisting of loss or gain of methylation, can be studied by site specific tests such as DNAm sensitive Multiplex Ligation-dependent Probe Amplification (MS-MLPA) or alternative site-specific technologies such as MS-PCR or MSqPCR.

An important aspect of BWS diagnostics is childhood tumor prediction since imprinting aberrations in the imprinting center IC1 (also called the *H19/IGF2* Differentially Methylated Region, DMR) are associated with high tumor risk whilst those in IC2 (*KCNQ1OT1* DMR) are not (Maas et al., 2016; Mussa et al., 2016; Brioude et al., 2018).

Genome wide methylation studies of patients with BWS and other imprinting disorder have discovered multiple aberrant imprinting loci associated with these disorders (MLID or multiple locus imprinting disturbance) (Sanchez Delgado et al., 2016; Fontana et al., 2018). DNAm arrays enabled us to add new aberrantly imprinted loci to this MLID pattern in BWS patients or even detect hypermethylation throughout the methylome in BWS cases (Krzyzewska et al., 2019b). It can therefore be concluded that the underlying (epi)genetic cause for an imprinting disorder can vary substantially between individuals and DNAm arrays enable personalized analyses of these patients. Limitations of DNAm arrays for diagnostic use are the costs of the technique. The Multi Locus MLPA kit (ME034) is more cost effective for the detection of MLID in diagnostics. This kit however is restricted to 7 imprinted regions associated with imprinting disorders (*PLAGL1, GRB10, MEST, H19, KCQ1OT1, MEG3, MEG8, SNRPN, PEG3, NESP55, GNAS-AS1, GNASXL, and GNAS A/B*). The clinical relevance for the DNAm defects at secondary loci is under investigation.

In the large majority of cases the loci involved in MLID, on top of the previously known disease associated loci, do not contribute to the clinical features of the primary imprinting disorder. Nonetheless, the diagnostic detection of MLID is becoming more and more relevant for routine diagnostic testing. Mutations in maternal effect genes (MEP) have been found in a small percentage of MLID patients and mutations in MEP genes are associated with reduced fertility and miscarriages (Begemann et al., 2018; Gheldof et al., 2019).

A more recent discovery is the existence of Whole Genome paternal Uniparental Disomy (GWpUPD) in an estimated 20% of BWS patients with a routine diagnosis of pUPD11. (Wilson et al., 2008; Kalish et al., 2013). DNAm arrays can be used to detect these genome wide DNAm changes of imprinted loci, but this is hampered by a low detection rate for low mosaicism. For routine diagnostics Multilocus Imprinting MLPA in combination with a SNP-array is recommended. Note that GWpUPD has been reported in association with tumor development at an age of > 7 years (reviewed in Postema et al., 2019), the age at which the increased risk of the development of childhood tumors associated with BWS falls back to the risk in the general population (Brioude et al., 2018).

The diagnostic consequence of all this is difficult to determine at present as more phenotype/(epi)genotype correlations are needed.

### DNAm Arrays as Diagnostic Tool/VUS Interpretation for Genetic Disorders, the Episign Test

Several hundred genes are associated with epigenetic programming. These genes code for writers-. erasers-, readers- or remodelers of the epigenome (chromatin). These genes are important for fetal and adult development and if mutated they cause various, predominantly developmental delay disorders as reviewed by Bjornsson, (2015), Weksberg et al. (2019).

For instance, mutations in the *NSD1* gene (encoding an epigenetic writer gene involved in histone methylation) cause Sotos syndrome. Mutations in *KMT2D* (also an epigenetic writer gene) cause Kabuki syndrome, as do mutations in *KDM6A* (an eraser gene involved in demethylation of histones) demonstrating the complexity of epigenetic programming. A well-known example of a reader gene causing disease once mutated is the *MECP2* gene causing Rett syndrome. Finally, *CHD7* is an example of a remodeler gene involved in CHARGEsyndrome.

**FIGURE 1:**
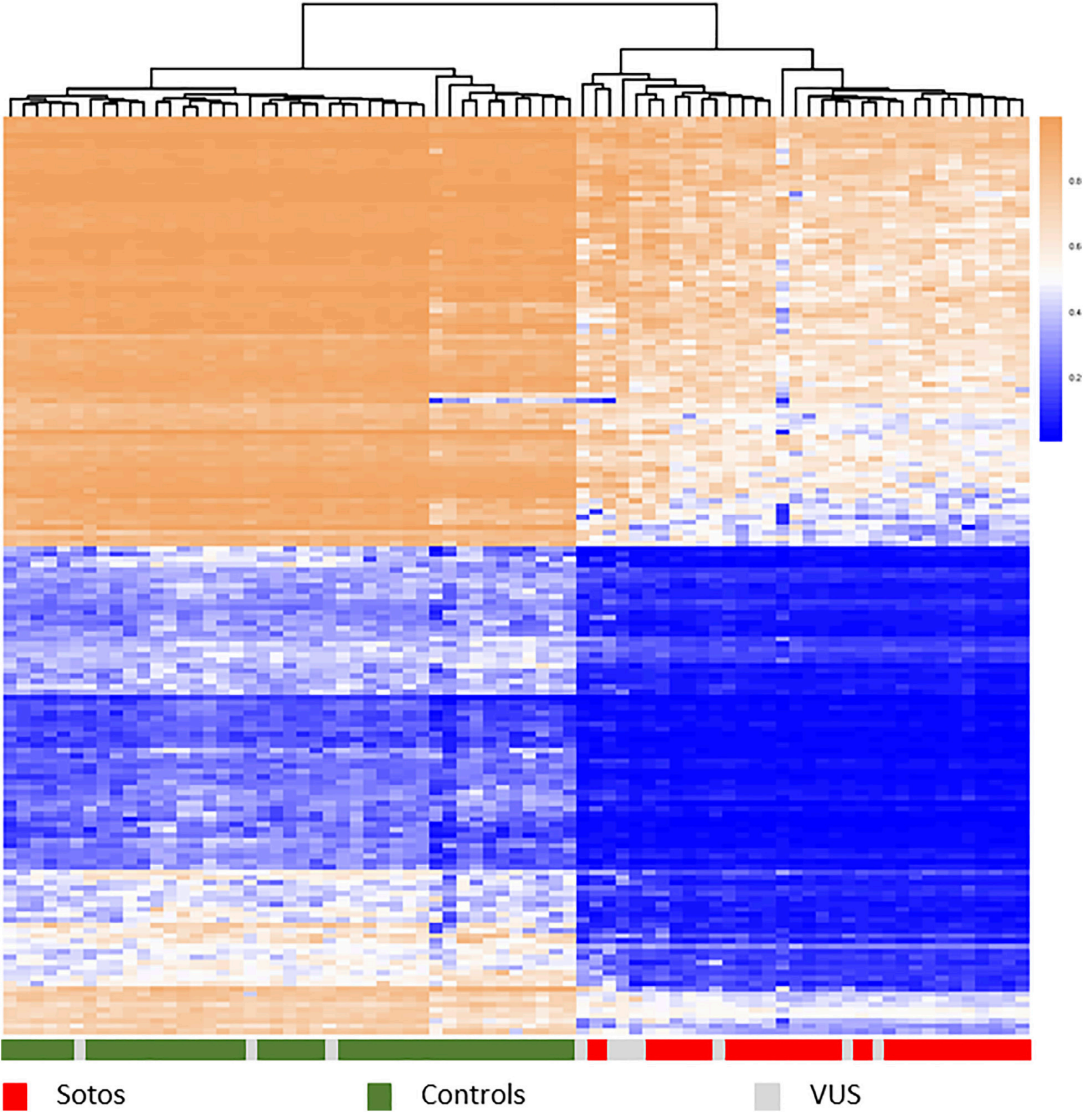
Hierarchical clustering heatmap showing different methylation profiles between Sotos syndrome patients (red) and control samples (green) using the top 1,000 most differentially methylated probes between these two groups. Methylation shown from 0 to 1. Patients carrying different types of a variant of unknown significance (grey) map either with controls or with cases, confirming the diagnosis Sotos syndrome in those clustered within the true cases. Samples: publicly available data set GSE74432 (62 samples, Choufani et al., 2015) and an Amsterdam UMC cohort of 15 samples.

Mutations in these genes lead to discrete aberrant genome DNAm patterns that are unique for specific disorders (Al-Jawahiri et al., 2022, Aref-Eshghi et al., 2017, 2020, Awamleh et al., 2022, Bend et al., 2019, Butcher et al., 2017, Cappuccio et al., 2020, Chater-Diehl et al., 2019, Cherik et al., 2022, Choufani et al., 2015a, 2020, Cuvertino et al., 2020, Foroutan et al., 2022, Haghshenas et al., 2016; Hood et al., 2016; Krzyzewska et al., 2019a, Levy et al., 2021a (2x), Rooney et al., 2021; Rots et al., 2021; Schenkel et al., 2017, 2018, 2021). and are detected relative to healthy controls and a growing list of various patient cohorts (Figure 1 shows an example of the methylation profile of Sotos syndrome patients relative to controls). In a single DNAm experiment, currently >60 disorders can be diagnosed and this number is growing annually (Levy et al., 2021b, website diagnostics laboratory*). This technology is available as a diagnostic test called Episign (developed by London Health Sciences, Ontario, Canada), which we have implemented in our diagnostic lab in the last few years. The latest version of this test, soon te be released, covers over 100 disorders.

The EpiSign test has proven to be very useful in diagnostics in those cases where the pathogenicity of a sequence variant is unknown. If this patient presents with a discrete DNAm pattern specific for the disorder studied, that information confirms the pathogenicity of the sequence variant to a high degree. In approximately 35% of patients referred for an EpiSign test because they carried a variant of unknown significance the (presumed) diagnosis was indeed confirmed. But also, in cases where whole exome sequencing did not reveal any possible causative variant, EpiSign can help to make a diagnosis. Around 10% of such cases displayed a DNAm signature that matched one of the known episignatures (Sadikovic et al., 2021). In some of these cases, subsequent targeted analysis of the gene(s) involved revealed the causative variant after all. Absence of a specific methylation profile however does not necessarily mean that a diagnosis is excluded nor that the variant is not pathogenic. For multiple genes it has been shown that different mutations within that gene can produce different DNAm signatures. In most cases this is accompanied by differences in phenotype, for instance the different episignatures for Nicolaides Baraitser syndrome and blepharophimosis intellectual disability syndrome, both caused by variants in the *SMARCA2* gene (Cappuccio et al., 2020). Other examples of genes with multiple signatures are *SRCAP* (Floating Harbor syndrome and DEHMBA) (Rots et al., 2021), *KMT2D* (Kabuki syndrome and CHARGE-like phenotype) (Cuvertino et al., 2020) and *KAT6B* (GTPTS and SBBYSS) (Aref-Eshghi et al., 2020). Interestingly, truncating variants in different parts of the ADNP gene do result in different signatures, however these patients do show only minimal differences in clinical presentation (Bend et al., 2019; Breen et al., 2020).

Due to the increasing number of human disorders that can be diagnosed with the EpiSign technology, these epigenetic tests have a high potential in genome diagnostics (Aref-Eshghi et al., 2020). Besides that, the same technology already enables the diagnostics of imprinted disorders and repeat sequence disorders. Moreover, episignatures may be detected and applied in human diseases associated to other genes than the aforementioned genes that code for chromatin associated proteins, as long as the disorder represents a specific methylation signature. Currently we are studying a number of such disorders such as type II diabetes mellitus (Meeks et al., 2019), obesity (Meeks et al., 2019) and Crohn’s disease (Li Yim et al., 2020), for which we detected dozens of novel disease associated loci. In addition, studies by others reported on numerous other examples of epigenetic changes associated with disease, summarized by Battram et al., 2021. These diseases are prevalent within the general population, generally reflect a heterogeneous phenotype and, obviously, a multi-factorial molecular basis, in accordance with the common variant - common disease hypothesis. The genetic component associated to these diseases often involves dozens to hundreds of genes which only partly can explain their heritability (Manolio et al., 2009). Moreover, such complex and common disorders typically involve a strong environmental component as well. Taken together, this suggests the presence of additional molecular components, for which epigenetics has been shown to be a good candidate. The multi factorial characteristic of these diseases challenges however the detection of clear epigenetic signatures tremendously. Moreover, most of these studies are based on surrogate tissue with respect to the affected tissue in disease, namely whole blood, which hampers any claims regarding causality of consequence effects. These limitations do however not necessarily prevent the detection of predictive epi signatures, in particular when statistical approaches address these adequately by means of in-depth phenotypic characterization (stratification) and application of machine learning methodology.

### Detection of Allele Specific Methylation

In case of (genome wide) significant DNAm association, complex traits generally represent aberrant methylation of loci on both alleles. Although such signal represents the average methylation signal of the set of cell (types) from which the DNA originated, allele specific methylation (ASM) is not uncommon as well. Two important ASM phenomena can be distinguished. Firstly, genomic imprinting, which represents the opposite DNAm of the paternal and maternal allele (Weksberg et al., 2019). Secondly, genotype specific methylation, which may comprise local genetic variation that affects DNAm at single and specific genomic positions at (or in vicinity of) the CpG dinucleotide site. Moreover, ASM also has been observed in relation to larger haplotypes, affecting multiple CpG dinucelotides. The average differential methylation signal as a consequence of allele specific DNAm differences is rather easy to detect (relative large effect size). For array based DNAm detection technology such as Illumina Infinium Methylation EPIC Beadchip array (850 k) genetic bias at the single base extension sites or probe sequence (Single Nucleotide Snips, SNPs) often is removed from the dataset, using Illumina’s manifest or dedicated software packages (Shan et al., 2016). However these SNPs can result in the additional detection of clustered DNAm profiles representing a proxy for genetic variance. A limited number of complex trait studies reported on mono allelic DNAm methylation, where differential methylation of the unchanged (CmpG) allele was observed while the other allele included the risk allelele (C > T) (Boks et al., 2016). Similarly, genomic imprinting defects can be detected using DNAm array profiles (Aref Esghi et al., 2020). Nevertheless, such imprinting aberrations often involve uniparental disomy (UPD) mosaicism. The detection of low mosaic (<10%) UPDs using array based technology remains limited. For in depth characterization of ASM, other technological platforms are available. Next generation sequencing (NGS) based methodology, such as whole genome or targeted bisulphite sequencing, can herein be applied. In combination with the limited read length of NGS fragments, its detection of allele specific methylation may be challenging. Recently, third generation sequencing (TGS) was introduced, where DNA (or RNA) can be sequenced directly omitting conversion of DNA and any amplification. Read length of sequenced fragments easily can cover thousands of base pairs. This way the generated genetic information can easily be used to define both alleles and haplotypes. Moreover, these technologies also enable the detection of DNA modifications as well. TGS methodology is a powerful new tool to detect ASM in relation to genetic variance or in relation to genomic imprinting and has already been applied in the field of oncogenomics and imprinting disorders (Gigante et al., 2019; McKelvey et al., 2020).

### DNAm Arrays and Environmentally Induced Disorders

#### Stress Related Disorders

Currently it is well known that trauma and stress influence our health. At the turn of this century, Vincent Fellitti and coworkers (Fellitti et al., 1998) studied the effect of child abuse and neglect (CAN) in a large cohort of American citizens. He found that these adversechildhood experiences (ACE) resulted in a broad spectrum of health problems later in life. Animal and human studies confirmed that epigenetic changes occurred due to these environmental triggers (Weaver et al., 2004; Mc Gowan et al., 2009; McGowan et al., 2009).

In our laboratory we have studied ACEs and post-traumatic stress disorder (PTSD) in adult and childhood cohorts (Krzyzewska et al., 2018; Nawijn et al., 2019; Ensink et al., 2021) with the Illumina methylation arrays and made the following observations:

- Not all individuals are susceptible to development of PTSD and genetic factors can be found that make individuals resilient to PTSD (Krzyzewska et al., 2018). Genes such as *CACNA1C, FKBP4, SDK1,* and *SKA2* seem to be associated with PTSD resilience. These genes are involved in stress regulation, neuron activity, psychiatric conditions, fear and suicide (Krzyzewska et al., 2018 and references therein).
- Epigenetic changes were predominantly found in genes that were associated with the neuronal dopamine system in the brain. Specific genes such as PAX8 demonstrated significant aberrant DNAm. This gene is a thyroid associated gene that might explain the disturbed thyroid function and sleeping disorders often found in individuals suffering from PTSD.
- Zooming in on PTSD in children we found that genes associated with immune response, neuronal development and stress response were aberrantly methylated compared to non-PTSD controls (healthy and trauma-exposed) (Ensink et al., 2021). Some of these differentially methylated loci correlated also with the activity in the prefrontal and hippocampus part of the brain as studied with MRI. The most significant genes were *OLFM3, GDF7,* and *TNXB*. Their cellular function fits well to the PTSD phenotype and brain changes.
- We are currently studying the effect of treatment (EMDR and cognitive therapy) on the epigenome of PTSD children. With machine learning approaches we aim to detect classifiers that enable predicting the response to PTSD intervention beforehand.
- Epigenetic changes due to child abuse and neglect may persist into adulthood. We are currently investigating this. In adults with PTSD we also noticed that aberrant DNAm of the oxytocin receptor gene was present in PTSD adult females, but not in males, which underlines differences in the etiology of PTSD between males and females as it has also been suggested between adults and children (Nawijn et al., 2019; Ensink et al., 2021). This might have consequences for treatment options.
- In a study conducted at our laboratory we analyzed the epigenome of a migrant population in Europe (e.g., residents of Ghana) and were able to associate perceived stress and ethnic discrimination to health expectation, cardiovascular risk, and diabetes (Chilunga et al., 2019; van der Laan et al., 2020). Other studies have reported a correlation between epigenetic markers and risk for cardiovascular disease. (Wada et al., 2021; Kraus et al., 2015; Agha et al., 2019).

In general, we conclude that such studies on environment-epigenome interactions demonstrate a discrete and measurable change at the molecular level. This provides a valuable tool to move forward in understanding these conditions and start diagnosing and treating them.

#### Alcohol Use During Pregnancy

Alcohol abuse during pregnancy may have devastating effects. In 2019 we demonstrated that children diagnosed with fetal alcohol spectrum disorder (FASD) presented numerous differentially methylated loci compared to non-alcohol *in utero* exposed controls (Cobben et al.,2019). Although many of those aberrations seem to have occurred randomly, several markers (annotated to *the GLI2, TNFRSG19, DTNA,* and *NECAB3* genes) were found to be significantly associated with the phenotype of FASD. The clinical diagnosis of FASD was based on the golden standard 4-digit score. Besides a range of different clinical features, the 4-digit score primarily takes into account the presence of alcohol exposure, facial abnormalities, brain abnormalities and growth abnormalities (Astley and Clarren, 2000). Currently we apply more sophisticated statistical models to search for a FASD specific DNAm signature and RNA expression studies are ongoing to look for eQTLs.

Apart from alcohol use, other toxic uptake, such as smoking, has a profound effect on the methylation status of the epigenome (Lee and Pausova, 2013). For this reason and together with age, it is generally corrected for as a confounder in studies with mDNA arrays on cohorts (Horvath, 2013).

#### Famine

Malnutrition during pregnancy can have a profound effect on health expectation for the fetus later in life especially if the famine occurred in early pregnancy (Painter et al., 2005; Roseboom et al., 2006; Roseboom et al., 2011; Veenendaal et al., 2013). In animal experiments, researchers described the influence of dietary constraints on the epigenome (Waterland and Jirtle, 2004; Dolinoy et al., 2007). In the Netherlands, two cohorts of women that were pregnant during the Dutch Famine in 1944–1945 and their offspring have been studied in Leiden and Amsterdam (Painter et al., 2005; Heijmans et al., 2008; Tobi et al., 2014).

Epigenetic differences have been linked to the Famine in these cohorts and some of these are already associated with human disease (Tobi et al., 2014). Detailed clinical information have been collected for the Amsterdam cohort consisting of the famine affected children, their mothers and their offspring. We are currently investigating the epigenetic differences and their correlation with clinical features.

#### Methylation Arrays and Cancer

Numerous publications are published describing epigenetic genomic loci, in particular regulatory regions, as markers for tumor development and progression. This field of study is too broad to cover in this manuscript. In short: some well-known examples are *MLH1* for colon cancer, *CDKN2A* and *HOXA9* for lung cancer, *GSTP1* for prostate cancer, *H19* for childhood tumors in Beckwith Wiedemann syndrome. Commercial tests are available to monitor markers for color cancer. DNA methylation profiling has significantly improved risk stratification in patients with adult brain tumors (Jaunmuktane et al., 2019). In our hospital, researchers are currently developing an epigenetic direct to consumer test that can be applied at home for HPV screening in relation to cervical cancer. Both array-based methylation tests as well as site specific methylation tests (e.g., promoter methylation or imprinting aberrations) are used in tumor diagnostics (for instance Jaunmuklane et al., 2019).

#### Treatment Prediction/Prognoses Through Machine Learning Approaches

While classical statistics, based on linear regression models, has been successfully applied to detect disease associated loci in omic based surveys (i.e., genome wide association studies), this methodology is far less suited to generate predictive outcomes. Predictive outcomes can involve prognosis and diagnosis of disease but can also be applied to detect signatures which can be used to predict the outcome of the disease treatment. The latter is a key aspect within personalized medicine and will improve patient care and decrease healthcare cost tremendously (Goecks et al., 2020). The basis of predictive algorithms is machine learning based methodologies, wherein the model is trained and tested applying tens to thousands of iterations. This approach yields a set of classifiers (loci) that *together* represent an optimal model for predictive purposes. Finally, the accuracy of such predictive model is then determined by its application in an independent (and blinded) cohort of cases and controls, wherein the specificity and sensitivity is validated. The performance success of machine learning methodology is dependent on the sample size. Moreover, these technologies generally work efficiently when the number of features (loci) is limited, which can be achieved by means of so-called *dimension reduction*. Within the field of molecular science, and in particular for genome diagnostics, both the sample size and number of features (omics) are often suboptimal. Fortunately, several methodologies and workflows have recently been developed that do enable machine learning application on smaller sample sets and high dimensional data. Currently the development of supervised (linear model) and unsupervised (variance) feature selection strategies have been shown to be successful. The methodology developed to detect DNAm signatures within the EpiSign panel is a good example, as previously described by Rooney et al. (2021). In brief, EpiSign signatures are based on a primary feature selection of often a limited but well characterized set of cases and a larger set of (standard) controls using a classic linear model. This analysis yields a limited set of individual features which is then validated using multiple iterations based on a “leave on out” strategy. Finally, the validated features are used in a *support vector machine* model, wherein specificity and selectivity are determined against every available control of distinct disease associated signature, yielding an accurate epigenetic signature. This strategy works for disorders associated with genes that code for chromatin associated proteins/enzymes. We are currently also applying this workflow on more complex diseases such as FASD and on predicting the response to PTSD therapy The fact that these two examples of complex and multi factorial disorders also yielded epi-signatures, indicates the great potential of machine learning technology. It is expected that future research will focus on further optimization of sample size and dimension reduction issues. Moreover, alternative approaches such as deep learning network analysis and inclusion of multi-omics (e.g. genome wide DNAm and gene expression profiles) based datasets within machine learning algorithms will be implemented (Demirel et al., 2021; Li et al., 2021; van der Vossen et al., 2021).

## Summary and Conclusion

Epigenetics increasingly plays an important role in medical genetics. Imprinting disorders have paved the way, but on top of this limited diagnostic package we now are able to diagnose an increasing number of human disorders with a single DNAm array test or with site-specific DNAm tests for discrete disorders and cancers.

More and more epigenetic biomarkers are discovered, enabling diagnostics and prognoses. Common multifactorial diseases caused by environmental factors are better understood with epigenetic studies aiding in preventing/predicting, diagnosing and prognosing these high frequency conditions such as cardiovascular disorders or stress related diseases. Our better understanding of epigenetic processes that lead to disease will also facilitate the availability of new drug targets. Epigenetic tests are very specific and often straightforward in interpretation provided the correct test is used for the disease studied (e.g., mDNA arrays have limitations in case of nucleotide expansion disorders or mosaic conditions. Site specific tests have a higher chance of missing relevant loci). Machine learning approaches will facilitate personalized treatment of patients. As discussed, the epigenetic array technology might guide treatment choice. The reversibility of epigenetic changes holds a promise for curing human diseases through a variety of interventions such as pharmacological-, cognitive/EMDR therapy or CRISPR/Cas9 repair. For all this, as always and above all we need good and extensive epigenetics databases of controls and well characterized patient cohorts.
